# Efficiency of complete omentectomy in patients with resectable gastric cancer: a meta‑analysis and systematic review

**DOI:** 10.1186/s12876-021-01921-3

**Published:** 2021-09-14

**Authors:** Akao Zhu, Guang Yin, Xinchun Liu, Wencheng Kong, Yu Zhang, Yuqiang Shan, Rongchao Ying, Jian Zhang, Chunhua Zhou

**Affiliations:** 1 Department of Gastrointestinal Surgery, Affiliated Hangzhou First People’s Hospital, Zhejiang University School of Medicine, Hangzhou, 31006 China; 2 Department of Surgical Oncology, Affiliated Hangzhou First People’s Hospital, Zhejiang University School of Medicine, Hangzhou, 31006 China

**Keywords:** Gastric cancer, Omentectomy, Overall survival, Complication, Meta-analysis

## Abstract

**Background:**

We performed a meta-analysis to investigate the efficacy of complete omentectomy (CO) in patients undergoing radical gastrectomy for gastric cancer.

**Methods:**

We conducted a literature search in PubMed, Web of Science, and the Cochrane Library databases for clinical research that compared CO with non-complete omentectomy (NCO). These articles were published prior to April 2021. Overall survival (OS) rates, relapse-free survival (RFS) rates, recurrence rates, operation times, estimates of blood loss, numbers of harvested lymph nodes, complications, and lengths of hospital stays were compared using relative risks (RRs) and weighted mean differences (WMDs). RevMan 5.3 software was used for statistical analysis.

**Results:**

Nine studies that included 3329 patients (1960 in the CO group) and 1369 in the NCO group comprised the analysis. The meta-analysis showed that CO was associated with a decreased 3-year OS rate (RR = 0.94, 95% CI 0.90–0.98, *P* = 0.005) and 5-year OS rate (RR = 0.93, 95% CI 0.88–0.98, *P* = 0.007). However, it was not associated with the 3-year RFS rate (RR = 0.97, 95% CI 0.90–1.04, *P* = 0.44), 5-year RFS (RR = 0.98, 95% CI 0.90–1.06, *P* = 0.60), or recurrence rate (RR = 1.17, 95% CI 0.95–1.45, *P* = 0.15) compared to the NCO group. For surgical-related outcomes, significant heterogeneity existed between the studies. Compared to the NCO group, CO was found to be associated with significantly more estimated blood loss (WMD = 250.90, 95% CI 105.90–396.28, *P* = 0.0007) and less harvested lymph nodes (WMD = − 3.59, 95% CI − 6.88, − 0.29, *P* = 0.03). Although, there was no significant difference in the surgical time (WMD = 15.93, 95% CI − 0.21, 32.07, *P* = 0.05). No statistically significant differences were observed in the rates of overall (*P* = 0.79) and major complications (*P* = 0.90), or the lengths of hospital stays (*P* = 0.11) between the two groups.

**Conclusions:**

Based on the available evidence, CO is not superior to NCO in terms of survival. CO is not recommended as a routine surgery for gastric cancer. Future well-designed high-quality RCTs are warranted.

**Supplementary Information:**

The online version contains supplementary material available at 10.1186/s12876-021-01921-3.

## Background

Gastric cancer (GC) poses a major threat to global health. It is estimated to be the fifth most commonly diagnosed cancer and the fourth leading cause of cancer-related mortality worldwide [1]. Although many treatment modalities, such as systemic chemotherapy, radiotherapy, immunotherapy, and targeted therapy have validated efficacy in GC, radical gastrectomy remains the mainstay of curative treatment for GC. Radical gastrectomy should be performed whenever possible [2]. However, the extent of radical gastrectomy for gastric cancer has not reached a consensus. For example, although commonly performed, the efficiency of complete omentectomy (CO) during radical gastrectomy has not yet been universally acknowledged [3–5]. The greater omentum is an apron-like fatty adipose tissue that extends from the stomach. It functions as a protective cushion and is responsible for peritoneal defenses [6]. The Japanese gastric cancer treatment guidelines recommend the removal of the greater omentum in standard gastrectomy for T3 or deeper tumors. The guidelines recommend the preservation of the omentum more than 3 cm away from the gastroepiploic artery for T1/T2 tumors [7]. To the best of our knowledge, there is only one meta-analysis that has explored the impact of omentectomy in patients with locally advanced gastric cancer [8]. This meta-analysis revealed that omentectomy had no significant impact on 5-y overall survival (OS) or 5-y recurrence-free survival (RFS). It included eight retrospective studies, including four studies in English, three studies in Japanese, and one study in Korean. It also included two studies that compared omentobursectomy and omentectomy. Moreover, since the literature search was conducted until December 2020, additional new studies have become available [9–11], including one randomized controlled trial [10].

Therefore, we designed and conducted this systematic review and meta-analysis to summarize the current evidence in the English literature on the clinical value of CO. This review was performed in terms of oncological outcomes, intraoperative safety, and postoperative recovery in GC patients undergoing gastrectomy.

## Methods

This systematic review was performed according to the Preferred Reporting Items for Systematic Reviews and Meta-Analyses (PRISMA) 2020 statement [12]. This study was not registered.

### Literature search strategy

We performed a systematic literature search in PubMed, Web of Science, and The Cochrane Library for eligible studies that investigated the efficacy of CO in patients who underwent gastrectomy for gastric cancer. The search strategy was (“omentum” OR “omentectomy” OR “omentum preservation” OR “omentum-preserving”) AND (“gastrectomy” OR “gastrostomy”) AND (“gastric cancer” OR “gastric carcinoma”). No language restrictions were used in the search strategy. The latest search was conducted on April 17, 2021.

### Study selection criteria and quality assessment

Studies that compared the outcomes of gastrectomy with CO and gastrectomy with non-CO (NCO) in patients with gastric cancer were included in the meta-analysis. The exclusion criteria were as follows: (1) non-human gastric cancer trials; (2) irretrievable data; (3) lack of comparison groups or lack of baseline data comparison; (4) evaluation of the correlation between bursectomy and outcomes in gastric cancer patients; (5) non-English articles; and (6) studies in the form of expert opinions, comments, or letters.

The quality of randomized controlled trials (RCTs) was assessed using the Cochrane Collaboration tool [13]. The quality of non-RCTs was assessed using the modified version of the Methodological Index for Non-randomized Studies (MINORS) [14]. A funnel plot analysis was performed to detect publication bias when more than ten studies were included.

### Outcome measures and data extraction

The primary outcomes of this meta-analysis were survival outcomes, including recurrence rates, OS rates, and the RFS rates of each eligible trial. The secondary outcomes included evaluation of CO in terms of surgical-related outcomes (operation time, estimated blood loss, and harvested lymph nodes) and postoperative recovery outcomes (overall complications, major complications, and length of postoperative hospital stays [LOS]).

The following data were independently extracted by two reviewers (AZ and YZ): first author, year of publication, country of investigators, study period, study design, sample size, study population characteristics, follow-up period, and primary and secondary outcomes. Inconsistencies between authors were resolved by discussion and arbitrated by a third reviewer (XL).

### Statistical analysis

The meta-analysis was performed using “Review Manager (RevMan). Version 5.3. Copenhagen: The Nordic Cochrane Centre, The Cochrane Collaboration, 2014”. When the mean or the standard deviation (SD) of an endpoint was not provided, it was calculated from the reported median, range, or interquartile range (IQR) if provided [15]. Moreover, survival outcome analyses were based on the extraction of unavailable data from the Kaplan–Meier curves. For dichotomous outcomes, risk ratios (RR) and the corresponding 95% confidence intervals (CI) were calculated using the Mantel–Haenszel (MH) method. For continuous outcomes, weighted mean differences (WMD) and corresponding 95% CI were calculated using the inverse variance method. *I*^*2*^ statistics were used to assess the statistical heterogeneity. *I*^*2*^ values of < 25, 25–50, and > 50% were considered low, moderate, and high heterogeneity, respectively. Given the heterogeneity of the tumors and patient characteristics, along with the diversity of the surgical approaches and techniques between studies, a random-effects model was used as the default for all statistical analyses. Subgroup analysis was performed for studies with a randomized or propensity score-matching (PSM) design. Sensitivity analysis was performed to evaluate the stability of the primary outcomes. The studies involved in the meta-analysis were deleted one by one to evaluate the influence of individual study data on the pooled effect estimate. Statistical significance was set at *P* < 0.05.

## Results

### Literature search results and characteristics of the included studies

**Fig. 1:**
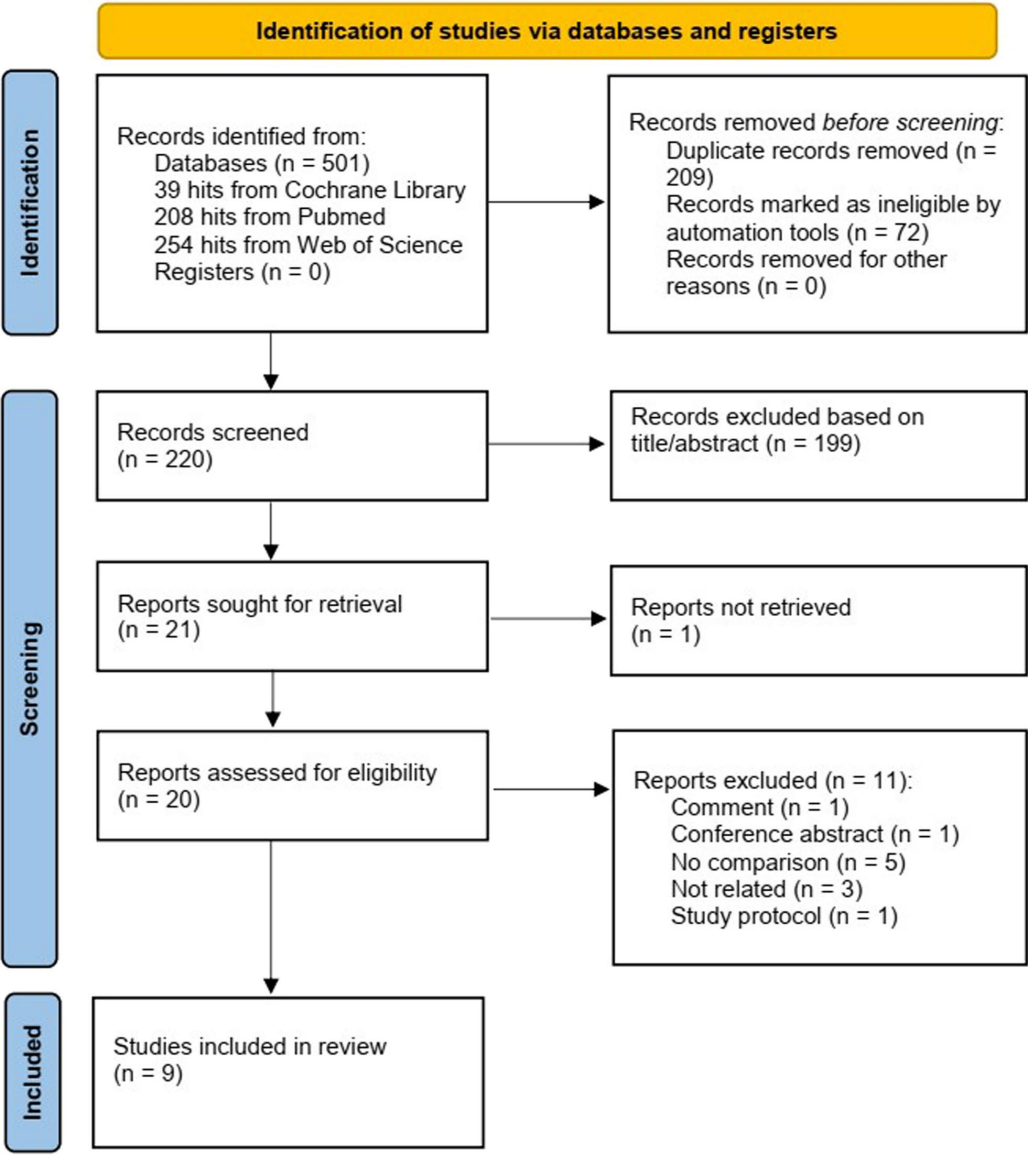
Flow chart showing study selection procedure

Figure 1 presents a flowchart detailing the study selection process. The search strategy initially extracted 501 items from the searched electronic databases (208 in PubMed, 39 in Cochrane Library, and 254 in Web of Science). After carefully removing duplications, screening the titles, abstracts, and full texts according to the inclusion and exclusion criteria, nine studies were included in the systematic review and meta-analysis. These studies were utilized to assess the efficacy of CO in gastrectomy for gastric cancer [4, 9–11, 16–20].

Of the nine studies, eight were conducted in East Asia (Korea or Japan), and one was conducted in the US. Of the nine studies, one was an RCT [10] and the remaining eight were retrospective comparative studies, with four using PSM. The study period of the included studies ranged from 2000 to 2018. The number of patients in each study ranged from 37 to 1116. Overall, 3329 patients were included in the meta-analysis, with 1960 patients in the CO group and 1369 patients in the NCO group.

The RCT quality assessment showed a low risk of bias in random sequence generation, low risk of bias in allocation concealment, high risk of bias in blinding of participants and personnel, unclear risk of bias in blinding of outcome assessment, high risk of bias in incomplete outcome data, unclear bias in selective reporting, and an unclear risk of other bias. Quality assessment of studies showed that all non-RCT studies had a score of ≥ 17 points out of 24 points, indicating that the studies were of high quality. The detailed baseline characteristics of the nine included studies are summarized in Table 1.

**Table 1:** Characteristics of the included studies

Authors	Country	Study design	Group	Sample size	Age (years)	Sex (M/F)	BMI (kg/m^2^)	Surgical approach (Open /MIS)	Procedure (DG/TG)	Pathological T stage	Lymphadenectomy extent	Score^b^
Seo et al. 2021	Korea	Retrospective PSM	CO	225	59 (49–70)	131/94	23.5 (21.1–25.6)	60/165	167/58	T3/T4a: 100/125	D1 + /D2: 25/200	20/24
			NCO	225	56 (49–67)	137/88	22.9 (21.0–24.9)	69/156	169/56	T3/T4a: 111/114	D1 + /D2: 22/203	
Murakami et al. 2021	Japan	Prospective RCT	CO	122	71 (30–90) ^a^	89/33	22.4 (14.8–31.8)	122/0	73/49	T1/T2/T3/T4: 20/21/42/39	D2: 122	-
			NCO	125	74 (45–89) ^a^	89/36	22.2 (14.5–32.1)	125/0	81/44	T1/T2/T3/T4: 31/21/31/42	D2: 125	
Sakimura et al. 2020	Japan	Retrospective PSM	CO	70	65.0 (37–90)	46/24	22.2 (15.8–30.3)	41/29	45/25	T0/T1/T2/T3/T4: 1/5/12/32/20	D1 + /D2 or more: 9/61	20/24
			NCO	70	66.5 (42–94)	48/22	22.4 (16.4–32.6)	25/45	44/26	T1/T2/T3/T4: 6/16/27/21	D1 + /D2 or more:14/56	
Ri et al. 2020	Japan	Retrospective PSM	CO	263	66.7 ± 11.0	176/87	22.4 ± 3.6	263/0	156/107	T1-2/T3-4: 47/216	≤ D1/D1 + /D2 or more: 11/146/106	22/24
			NCO	263	65.7 ± 12.9	181/82	22.5 ± 3.4	263/0	151/112	T1-2/T3-4: 48/215	≤ D1/D1 + /D2 or more: 8/146/109	
Young et al. 2020	USA	Retrospective	CO	90	69.5 (62–77)	62/28	27.4 ± 6.1	NA	TG:25, STG: 31	T3 or T4: 41	NA	18/24
			NCO	381	68 (58–76)	217/164	26.2 ± 5.3	NA	TG:99, STG: 140	T3 or T4: 184	NA	
Kim et al. 2014	Korea	Retrospective	CO	80	60.9 ± 11.2	56/24	NA	0/80	61/19	T2/T3: 28/52	D1 + /D2: 2/78	19/24
			NCO	66	62.2 ± 11.0	50/16	NA	0/66	24/12	T2/T3: 37/29	D1 + /D2: 5/61	
Hasegawa et al. 2013	Japan	Retrospective PSM	CO	98	69.0 (40–91)	72/26	NA	98/0	52/46	T2/T3/T4a: 30/34/34	D1 + /D2 or more:12/86	17/24
			NCO	98	68.7 (45–91)	72/26	NA	84/14	61/37	T2/T3/T4a: 34/30/34	D1 + /D2 or more:13/85	
Kim et al. 2011	Korea	Retrospective	CO	20	58.2 ± 9.5	17/3	23.3 ± 2.3	20/0	20/0	T1/T2: 20/0	≤ D1 + β/ > D2: 13/7	17/24
			NCO	17	58.6 ± 10.1	11/6	23.6 ± 2.7	17/0	17/0	T1/T2: 16/1	≤ D1 + β/ > D2: 15/2	
Ha et al. 2008	Korea	Retrospective	CO	992	57.0 ± 11.3	681/311	NA	NA	TG: 75, Others: 917	early gastric cancer	NA	19/24
			NCO	124	56.3 ± 11.3	87/37	NA	NA	TG: 25, Others: 99	early gastric cancer	NA	

CO, complete omentectomy; NCO, non-complete omentectomy; BMI, body mass index; DG, distal gastrectomy; TG, total gastrectomy; STG: subtotal gastrectomy; PSM, propensity score matching; RCT, randomized controlled study; MIS, minimally invasive surgery; NA, not available

^a^Data are shown as median (range); ^b^MINORS score for non-RCTs

### Meta-analysis of outcomes

#### Survival outcomes

**Fig. 2:**
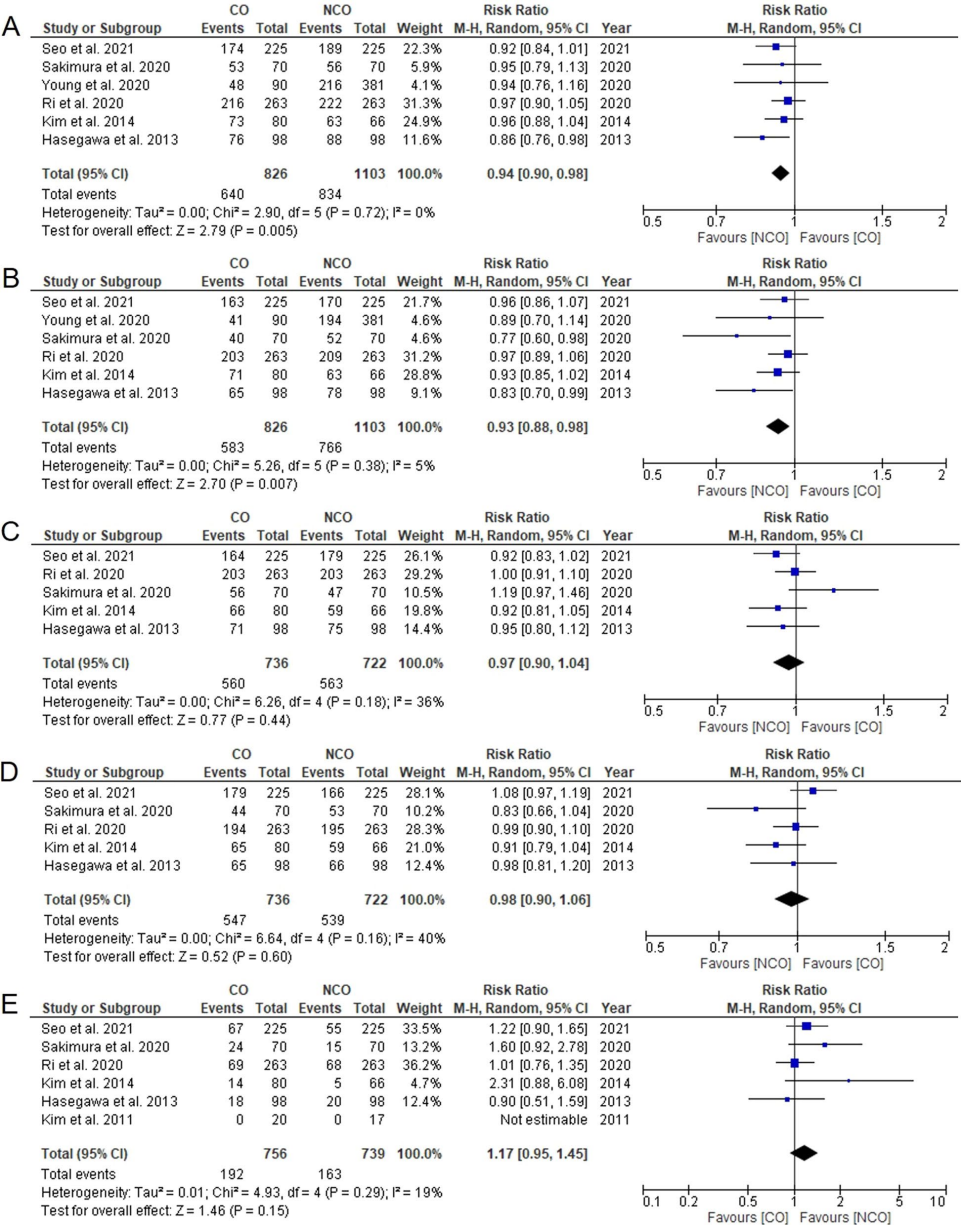
Meta-analysis comparing **A** 3-year overall survival rate, **B** 5-year overall survival rate, **C** 3-year relapse-free survival rate, **D** 5-year relapse-free survival, **E** recurrence rate. CO, complete omentectomy; NCO, non-complete omentectomy

Six studies reported data on 3-year-and 5-year OS with low heterogeneities [4, 9, 11, 17, 18, 20]. The meta-analysis showed that the NCO group had a better 3-year OS rate than the CO group (RR = 0.94, 95% CI 0.90–0.98, *P* = 0.005, *I*^2^ = 0%, Fig. 2A). The NCO group also had a better 5-year OS rate than the CO group (RR = 0.93, 95% CI 0.88–0.98, *P* = 0.007, *I*^2^ = 5%, Fig. 2B).

Five studies reported data on 3-year and 5-year RFS with moderate heterogeneities [4, 11, 17, 18, 20]. The meta-analysis showed that there was neither a significant difference in the 3-year RFS rate (RR = 0.97, 95% CI 0.90–1.04, *P* = 0.44, *I*^2^ = 36%, Fig. 2C) nor in the 5-year RFS rate (RR = 0.98, 95% CI 0.90–1.06, *P* = 0.60, *I*^2^ = 40%, Fig. 2D) between the CO and NCO groups.

Six studies reported data on recurrence with low heterogeneity [4, 11, 17–20]. No statistically significant difference in recurrence rate was observed between the CO and NCO groups (RR = 1.17, 95% CI 0.95–1.45, *P* = 0.15, *I*^2^ = 19%, Fig. 2E).

#### Surgical-related outcomes

**Fig. 3:**
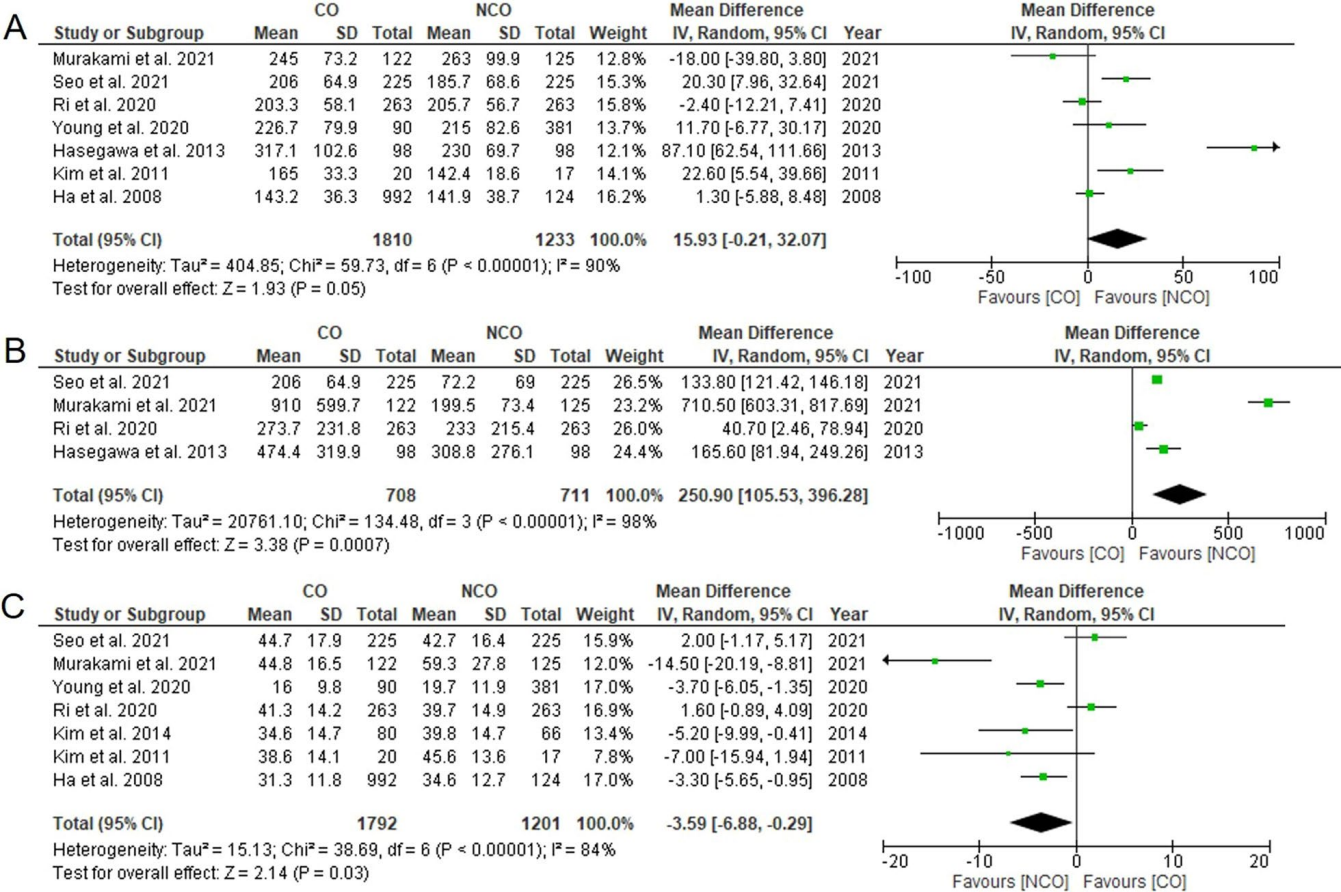
Meta-analysis comparing **A** operation time, **B** estimated blood loss, **C** harvested lymph nodes. CO, complete omentectomy; NCO, non-complete omentectomy

Six studies reported data on surgical time with high heterogeneity [4, 9–11, 16, 17, 19]. The meta-analysis revealed that the operation time of the CO group was longer than that of the NCO group. However, the difference was not significant (WMD = 15.93, 95% CI − 0.21 to 32.07, *P* = 0.05, *I*^2^ = 90%, Fig. 3A).

Four studies reported data on estimated blood loss with high heterogeneity [4, 10, 11, 17]. The meta-analysis revealed that the CO group was associated with significantly more estimated blood loss than the NCO group (WMD = 250.90, 95% CI 105.90–396.28, *P* = 0.0007, *I*^2^ = 98%, Fig. 3B).

Two studies did not report the types of lymphadenectomy. There was heterogeneity in the types of lymphadenectomy among the remaining studies (Table 1). Nevertheless, seven studies reported data on the number of harvested lymph nodes with high heterogeneity [4, 9–11, 16, 18, 19]. The meta-analysis revealed that CO group had significantly fewer harvested lymph nodes than NCO group (WMD = − 3.59, 95% CI − 6.88, − 0.29, *P* = 0.03, *I*^2^ = 84%, Fig. 3C).

#### Postoperative recovery outcomes

**Fig. 4:**
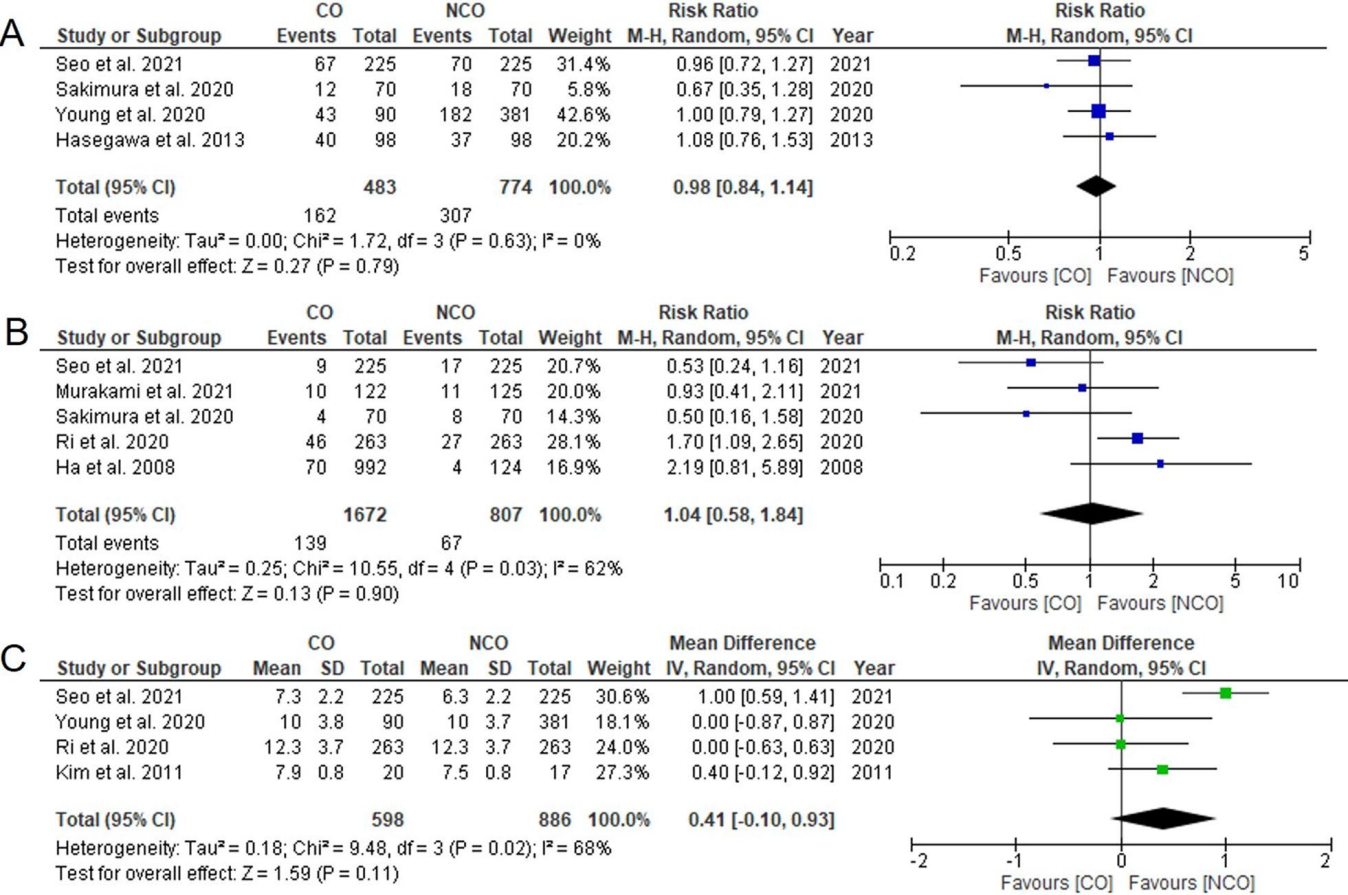
Meta-analysis comparing **A** overall complications, **B** major complications, **C** length of postoperative hospital stay. CO, complete omentectomy; NCO, non-complete omentectomy

Overall and major complications were reported in 4 [9, 11, 17, 20] and five studies [4, 10, 11, 16, 20], respectively. Meta-analysis revealed that there was no significant difference in overall (RR = 0.98, 95% CI 0.84–1.14, *P* = 0.79, *I*^2^ = 0%, Fig. 4A) or major complication rates (RR = 1.04, 95% CI 0.58–1.84, *P* = 0.90, *I*^2^ = 62%, Fig. 4B) between the two groups.

Four studies mentioned the length of postoperative hospital stays with high heterogeneity [4, 9, 11, 19]. The length of hospital stays was similar between the two groups (WMD = 0.41, 95% CI − 0.10 to 0.93, *P* = 0.11, *I*^2^ = 68%, Fig. 4C).

### Subgroup analysis

**Fig. 5:**
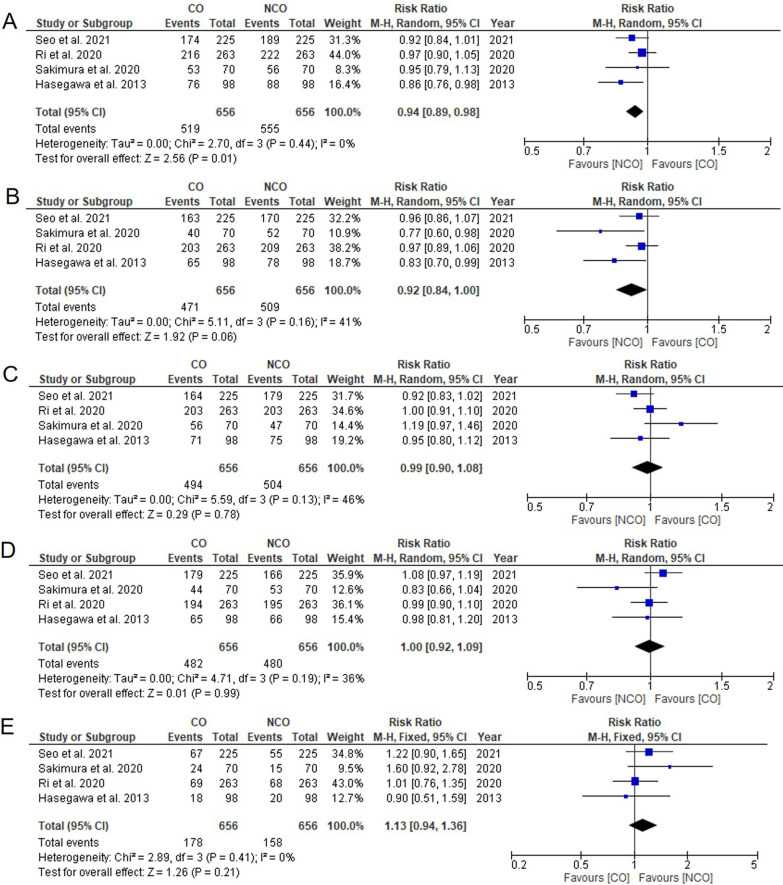
Subgroup meta-analysis for studies with PSM or randomized designs comparing **A** 3-year overall survival rate, **B** 5-year overall survival rate, **C** 3-year relapse-free survival rate, **D** 5-year relapse-free survival, **E** recurrence rate. CO, complete omentectomy; NCO, non-complete omentectomy

Subgroup analyses were performed for studies with PSM or randomized designs [4, 10, 11, 17, 20]. The meta-analysis confirmed that NCO was associated with a better 3-year OS rate (RR = 0.94, 95% CI 0.89–0.98, *P* = 0.01, *I*^2^ = 0%, Fig. 5A) and a lower estimated blood loss (WMD = 250.90, 95% CI 105.90–396.28, *P* = 0.0007, *I*^2^ = 98%, Additional file 1: Fig. S1). However, it was not associated with the other remaining outcomes (Fig. 5, Additional files 1 and 2: Figs. S1 and S2).

### Sensitivity analysis and publication bias

To perform sensitivity analysis, studies were excluded in turn to assess whether individual studies influenced the pooled RRs of the outcomes. For every meta-analysis of the primary outcomes and postoperative recovery outcomes, the pooled RRs were similar after each study was excluded. This verified the stability of the meta-analysis. For every meta-analysis of the operation-related outcomes, the pooled RRs were significantly changed after the selective studies were excluded. Publication bias was not assessed as fewer than ten studies were included in the meta-analysis.

## Discussion

This meta-analysis systematically investigated the effects of CO on radical gastrectomy for gastric cancer. Our study showed that CO did not improve survival compared to NCO. In contrast, CO was associated with significantly worse 3-year and 5-year OS. Although, there was no significant difference in the 3-year and 5-year RFS or recurrence rates. Moreover, CO was associated with more estimated blood loss and fewer harvested lymph nodes. There was no statistically significant difference in the postoperative recovery, in terms of overall complications, major complications, and LOS between the CO and NCO groups.

D2 lymphadenectomy represents the gold standard for treating advanced gastric cancer [21]. Laparoscopic gastrectomy is widely accepted as an alternative to open gastrectomy [22–24]. However, the extent of resection remains to be determined. Previously, bursectomy, which removes the anterior membrane of the transverse mesocolon and the peritoneal lining covering the pancreas with a total omentectomy, has been recommended as an essential part of complete radical gastrectomy [25]. However, this has been challenged. Recently, Xiong et al. performed a meta-analysis of 15 studies with 4858 patients to investigate the safety and efficiency of bursectomy during radical gastrectomy for patients with gastric cancer [26]. They found that although bursectomy was associated with a prolonged operative time and more intraoperative blood loss, it was not superior to non-bursectomy. This was in terms of oncological outcomes, such as the number of dissected lymph nodes, survival rates, and recurrence rates. Therefore, Xiong et al. concluded that bursectomy should not be a routine surgery for resectable cT3 or cT4a gastric cancer. This was supported by a high-quality RCT [27].

The aim of CO was similar to that of bursectomy, with the removal of concurrent micrometastases and potential sites of recurrence. However, the prevalence and significance of concurrent omental micrometastases remains controversial. One study found that 10% of gastrectomy patients harbored tumor deposits or lymph node metastases in the greater omentum [5]. Moreover, factors predicting omental tumor involvement could not be identified. Therefore, omentectomy should be the standard gastrectomy for patients with gastric cancer [5]. Meanwhile, other studies found that metastases in the greater omentum occurred in only 1.6% of GC patients who underwent gastrectomy [3, 28]. These were correlated significantly with non-radical features and advanced disease, indicating stage IV disease and a poor prognosis [11, 29]. In another study, Metwally et al. compared the survival of gastric cancer patients with/without tumor infiltration in the omentum. They found that omental infiltration was not associated with overall or disease-free survival [30].

In this meta-analysis, the included studies consistently found no significant survival benefit for CO. Moreover, a meta-analysis of all included studies found that CO is associated with a decreased 3-year OS and 5-year OS. Subsequent subgroup analysis for randomized or PSM studies confirmed that CO was associated with a decreased 3-year OS, but not with 5-year OS. This is in line with the findings of Ishizuka et al. [8]. In addition, NCO is less challenging, with less estimated blood loss. Although one study by Olmi et al. [31] found that omentum preservation was associated with a lower incidence of recurrence and a lower incidence of complications than in patients with omentectomy, these effects were not observed in this meta-analysis. However, it is of note that omental preservation may have specific complications, such as omental infarction and trocar herniation. This may cause abdominal pain [32, 33]. This meta-analysis also found that NCO was associated with a higher LN yield, which is contrary to intuition. One potential reason might be that even in omentum preservation, lymph nodes along the gastroepiploic arcade could be dissected completely [11]. However, a smaller omental specimen might compel the pathologist to check the lymph nodes more cautiously. This would result in a higher LN yield. This is more likely to be associated with different types of lymphadenectomies between studies.

This study had several limitations. First, the number of eligible studies was limited and some outcomes of interest were not reported in all eligible studies. Furthermore, some outcomes suffer from moderate to high heterogeneity between the two groups. The types of lymphadenectomy and the harvested lymph nodes are examples, thus decreasing the power of this meta-analysis. Second, most included studies were from Japan and Korea, which may limit the application of the results in Western populations. Third, although it is likely that omentectomy might be useful for patients with T3/4 stage gastric cancers, it was impossible to perform a subgroup analysis with few studies focusing on the effect of CO in this subgroup of patients. Despite these limitations, the results of the present meta-analysis challenged the justification for CO. Omentum preservation could be a better choice than CO with regard to oncological outcomes and surgical effort.

## Conclusions

In conclusion, CO did not benefit survival, operative, or recovery outcomes when compared to NCO. Based on the available evidence, CO is not recommended as a standard procedure for resectable gastric cancer. Future well-designed, high-quality RCTs are warranted to clarify the efficacy of CO in radical gastrectomy, especially in cT3 or cT4 gastric cancer.

## Supplementary Information

**Additional file 1**. **Fig. S1.** Subgroup meta-analysis for studies with PSM or randomized designs comparing **A** operation time, **B** estimated blood loss, **C** harvested lymph nodes. CO, complete omentectomy; NCO, non-complete omentectomy.

**Additional file 2.Fig. S2.** Subgroup meta-analysis for studies with PSM or randomized designs comparing **A** overall complications, **B** major complications, **C** length of postoperative hospital stay. CO, complete omentectomy; NCO, non-complete omentectomy.

## Data Availability

The original data used in the study are all included in the article, further inquiries can be directed to the corresponding author.

## Abbreviations

GC: Gastric cancer
CO: Complete omentectomy
OS: Overall survival
RFS: Relapse-free survival
PRISMA: Preferred Reporting Items for Systematic Reviews and Meta-Analyses
NCO: Non- complete omentectomy
RCT: Randomized controlled trial
MINORS: Methodological Index for Non-randomized Studies
LOS: Postoperative hospital stays
SD: Standard deviation
RR: Risk ratios
MH: Mantel–Haenszel
WMD: Weighted mean differences
CI: Confidence intervals
PSM: Propensity score-matching
