# Supplementary figures and images for: Efficiency of complete omentectomy in patients with resectable gastric cancer: a meta‑analysis and systematic review
Source: BMC Gastroenterol. 2021 Sep 14;21:346. doi: 10.1186/s12876-021-01921-3 (PMC8439052; doi:10.1186/s12876-021-01921-3)

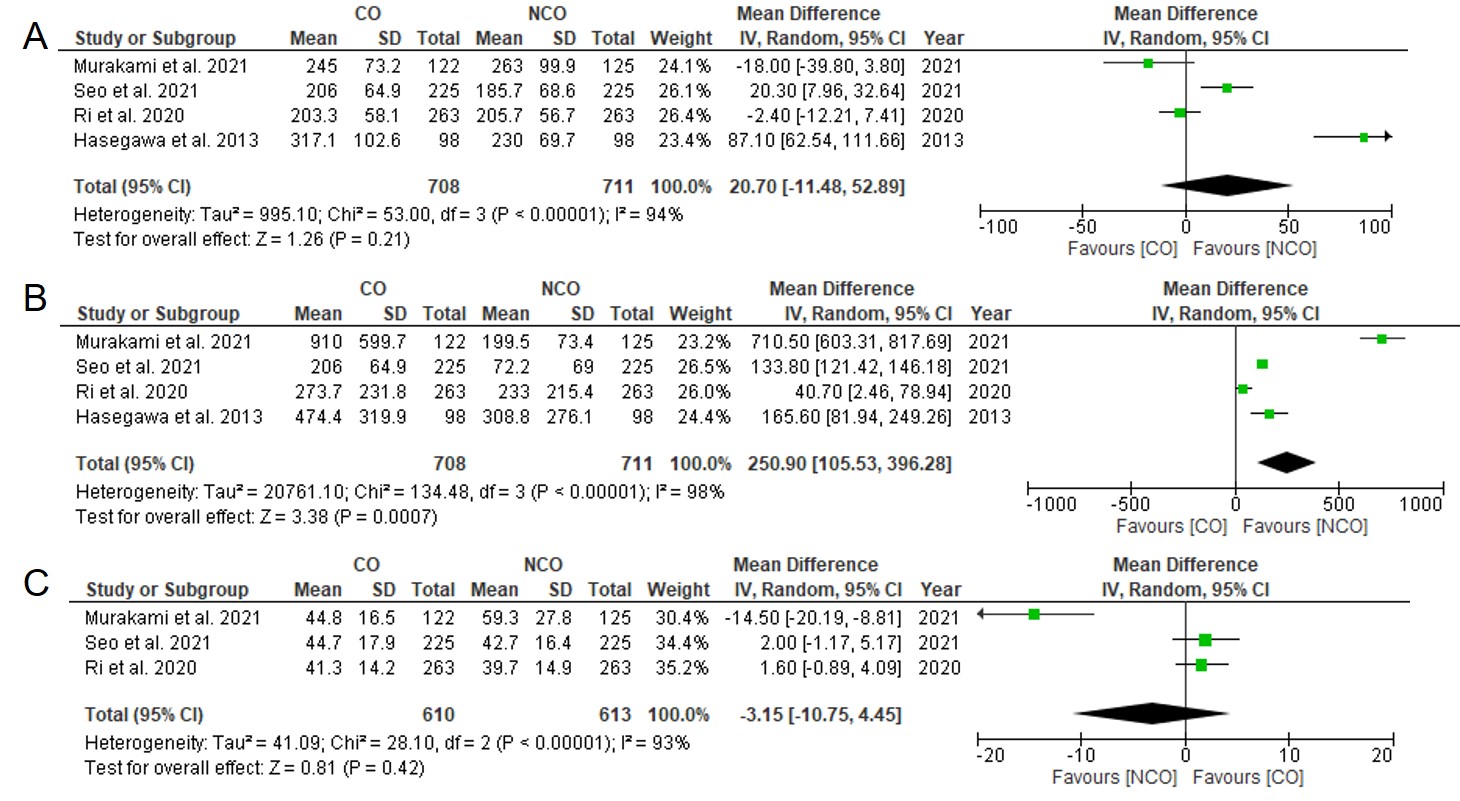

Supplement: Supplementary file 1 — Additional file 1. Fig. S1. Subgroup meta-analysis for studies with PSM or randomized designs comparing A operation time, B estimated blood loss, C harvested lymph nodes. CO, complete omentectomy; NCO, non-complete omentectomy. [file 12876_2021_1921_MOESM1_ESM.jpg]

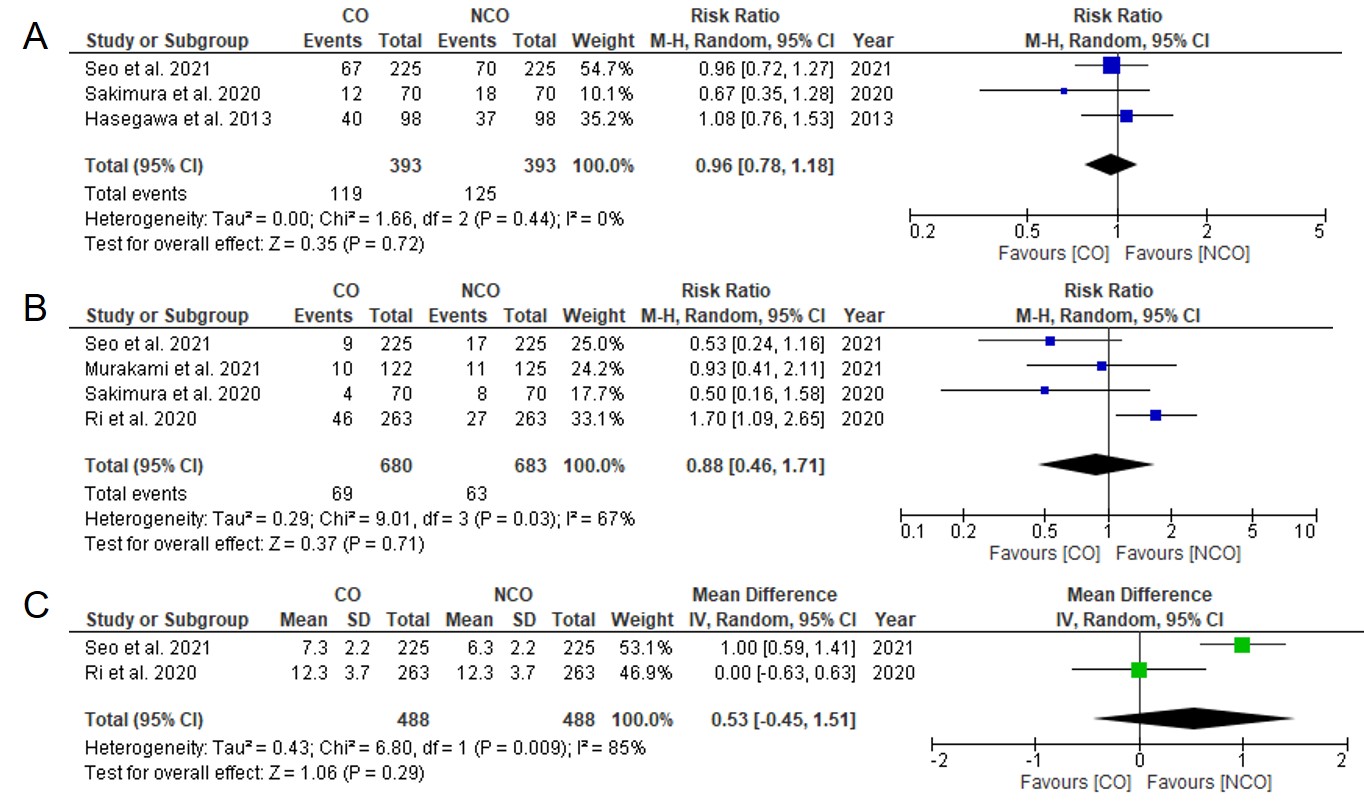

Supplement: Supplementary file 2 — Additional file 2.Fig. S2. Subgroup meta-analysis for studies with PSM or randomized designs comparing A overall complications, B major complications, C length of postoperative hospital stay. CO, complete omentectomy; NCO, non-complete omentectomy. [file 12876_2021_1921_MOESM2_ESM.jpg]
